# E2VIDX: improved bridge between conventional vision and bionic vision

**DOI:** 10.3389/fnbot.2023.1277160

**Published:** 2023-10-26

**Authors:** Xujia Hou, Feihu Zhang, Dhiraj Gulati, Tingfeng Tan, Wei Zhang

**Affiliations:** ^1^School of Marine Science and Technology, Northwestern Polytechnical University, Xi'An, China; ^2^Siemens EDA, Munich, Germany

**Keywords:** image reconstruction, deep learning, dynamic vision sensor, event camera, image classification, object detection, instance segmentation

## Abstract

Common RGBD, CMOS, and CCD-based cameras produce motion blur and incorrect exposure under high-speed and improper lighting conditions. According to the bionic principle, the event camera developed has the advantages of low delay, high dynamic range, and no motion blur. However, due to its unique data representation, it encounters significant obstacles in practical applications. The image reconstruction algorithm based on an event camera solves the problem by converting a series of “events” into common frames to apply existing vision algorithms. Due to the rapid development of neural networks, this field has made significant breakthroughs in past few years. Based on the most popular Events-to-Video (E2VID) method, this study designs a new network called E2VIDX. The proposed network includes group convolution and sub-pixel convolution, which not only achieves better feature fusion but also the network model size is reduced by 25%. Futhermore, we propose a new loss function. The loss function is divided into two parts, first part calculates the high level features and the second part calculates the low level features of the reconstructed image. The experimental results clearly outperform against the state-of-the-art method. Compared with the original method, Structural Similarity (SSIM) increases by 1.3%, Learned Perceptual Image Patch Similarity (LPIPS) decreases by 1.7%, Mean Squared Error (MSE) decreases by 2.5%, and it runs faster on GPU and CPU. Additionally, we evaluate the results of E2VIDX with application to image classification, object detection, and instance segmentation. The experiments show that conversions using our method can help event cameras directly apply existing vision algorithms in most scenarios.

## 1. Introduction

Robots have become indispensable in modern society, capable of replacing manual labor to execute repetitive and hazardous tasks, thereby enhancing production efficiency and quality while reducing production costs (Jing et al., 2022). Various research studies in the field of robotics are continuously carried out by Bing et al. (2022, 2023a,b). In the realm of robotics, computer vision plays a pivotal role in tasks such as robot navigation, perception, and decision-making. Most commonly used camera sensors include CMOS (Sukhavasi et al., 2021), CCD (Adam et al., 2019), and RGBD (Liu et al., 2022) cameras, all of which share a standard parameter: frame rate. These cameras capture images at consistent time intervals, synchronizing their data acquisition. However, they often yield suboptimal results in high-speed motion scenes or environments with inadequate lighting conditions due to their imaging principles. To solve this problem, researchers (Posch et al., 2014) have developed event cameras, sometimes called dynamic vision sensor (DVS). Instead of capturing images at a fixed frame rate, event cameras capture “events”, which are triggered when the cumulative brightness change of a pixel reaches a certain threshold. An event has three elements: timestamp, pixel coordinate, and polarity. Therefore, an event expresses when (i.e., time), at which pixel, an increase or decrease in brightness occurs. Event camera imaging principle guarantees that as long as the brightness change exceeds the threshold value, there will be an output, and it requires small bandwidth. In other words, if there are objects moving very fast in the camera's field of view, it will generate multiple events per second. If there is no object motion or brightness change, there are no events generated. At the same time, since the event camera is better at capturing the brightness change, it performs equally in dark and intense light scenes. Therefore, event cameras have the advantages of low latency, high dynamic range (140 vs. 60 dB), and low power consumption and are not affected by motion blur compared with regular frame-based cameras (Gallego et al., 2020).

**Figure 1:**
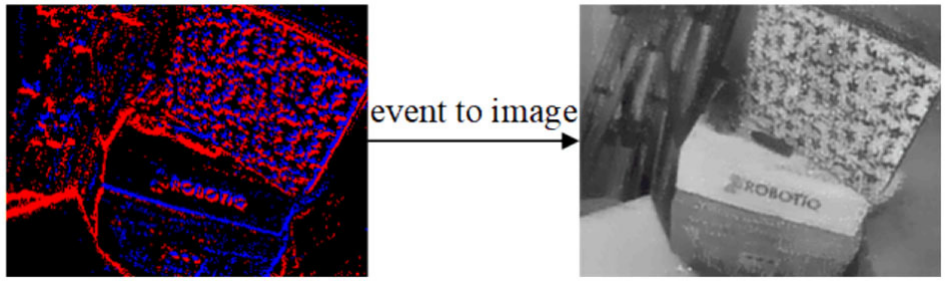
A schematic of an image generated from the event stream (shot in high speed motion scene), with blue for negative polarity and red for positive polarity.

Although an event camera has been successfully used in SLAM (Vidal et al., 2018), human detection (Xu et al., 2020), and other fields (Zhou et al., 2018; Perot et al., 2020), the output format of an event camera is far from the familiar camera output format. Therefore, it does not easily lend itself to practical applications. Compared with events alone, reconstructing images from events (as shown in Figure 1) provides a compact representation of the latest available data and enables the application of traditional computer vision to event cameras. In contrast to raw events, images possess a natural interpretability for humans and encompass a broader spectrum of information. Additionally, the reconstructed image offers a synthesis of several advantageous attributes, including high temporal resolution, spatial interpretability, and robust resistance to interference. Consequently, traditional vision algorithms can be seamlessly employed with reconstructed images, eliminating the necessity for the redesign of additional algorithms when integrating event cameras into applications.

In the early days of this field, researchers derived the reconstruction formula by modeling the imaging principle of event cameras (Brandli et al., 2014; Munda et al., 2018; Scheerlinck et al., 2018). However, due to the sensor noise, the reconstruction was far from ground truth images. With advent of the powerful deep learning methodology in recent years, we are able to improve the reconstruction and the results converge to the ground truth (Rebecq et al., 2019a,b; Wang et al., 2019; Scheerlinck et al., 2020; Cadena et al., 2021). While advancements in reconstruction techniques have led to improvements, the utilization of deep neural networks often necessitates substantial time and computational resources. Consequently, their application to edge or mobile devices is constrained. Furthermore, the network architectures developed using some of the current methods do not readily scale down to these resource-constrained devices. To address this challenge, this study proposes E2VIDX, a faster and stronger neural network for image reconstruction. By changing the feature fusion, the network is further optimized by using group convolution and sub-pixel convolution. Simultaneously, this study proposes a simplified loss function to counter the excessive number of parameters. Furthermore, the effectiveness of the proposed E2VIDX is demonstrated by applying it to various high-level vision tasks, including image classification, object detection, and instance segmentation, using the reconstructed images as input data. These applications illustrate the practical utility of E2VIDX in real-world scenarios.

In summary, the main contributions of this study are as follows:

- This study proposes an improved event reconstruction method: E2VIDX. On comparing with the state-of-the-art, not only E2VIDX outperforms on the three evaluation indicators but it also has shorter reconstruction time.
- Ablation study is presented to prove the effectiveness of the proposed module.
- Designed high-level vision tasks completed to qualitatively and quantitatively evaluate the reconstructed images obtained using E2VIDX.

## 2. Related work

In the domain of event processing, the mainstream image reconstruction algorithms can be divided into two types, namely, asynchronous event processing and synchronous batch processing.

### 2.1. Asynchronous event processing

The idea is to use the sparsity of events; as soon as the event arrives, the new information is integrated into the existing state for updating. Since the information contained in a single event is very little, one of the focuses of asynchronous algorithm research is how to fuse the existing information with the current event, which also requires that the algorithm needs an image or waits enough time when initializing. Brandli et al. (2014) first proposed using event streams for image reconstruction. They used the complementarity of regular cameras and event cameras to insert events marked with thresholds between two consecutive frames. The threshold is determined by the difference in event summary between two consecutive frames. This method has low computational overhead and can run in real-time using only a CPU, but it must require frame-based images as dense as possible. Reinbacher (Munda et al., 2018) treat the image reconstruction problem as an energy minimization problem, model the noise based on the generalized Kullback–Leibler divergence to prevent noise accumulation, and define the optimization problem as an event flow pattern containing timestamps. Finally, it used the variational method to optimize. Scheerlinck et al. (2018) proposed to use complementary filters to reconstruct intensity images from asynchronous events, with an option to incorporate information into image frames. Complementary filters perform temporal smoothing but do not perform spatial smoothing, which dramatically improves the computational efficiency and significantly improves the reconstruction speed.

Although the above methods, based on mathematical and physical modeling, are reliable in theory, cumulative error of the reconstructed image increases with time because the sensor noise is affected by temperature, humidity, and electrical devices. At the same time, another non-negligible problem is that the contrast threshold of the event camera is different at each pixel and changes over time. Therefore, methods based on asynchronous event processing are limited in their usage scenarios.

### 2.2. Synchronous batch processing

Batch image reconstruction aims to reconstruct an image or video by considering a batch of events rather than a single event, primarily using popular machine learning methods for modeling. To deal with how the event stream is fed into the network, Wang et al. (2019) proposed two batch processing methods, namely, time-based event stream input and event number-based input. Finally, they successfully used the Conditional Generative Adversarial Network (CGAN) to reconstruct and obtain the image with high dynamic range and no motion blur. E2VID, proposed by Rebecq et al. (2019a,b) is the first method to combine convolutional neural network (CNN) and recurrent neural network (RNN) for image reconstruction. It achieves end-to-end video reconstruction with supervised learning from simulated event data, resulting in images with high resolution in time and high-speed motion scenes. Considering the low latency of events, Scheerlinck et al. (2020) modified E2VID, by replacing the original U-Net (Ronneberger et al., 2015) structure with a stacked structure, and obtained FireNet with fewer parameters and faster operation but with almost the same accuracy. E2VID uses a recurrent neural network to fuse previous information, hence fewer frames are needed to initialize at the beginning stage of reconstruction. SPADE-E2VID (Cadena et al., 2021) adds a SPADE module (Park et al., 2019) to solve this problem, significantly reducing the initialization time. At the same time, a loss function without temporal consistency is proposed to speed up the training speed.

Image reconstruction based on deep learning has made significant progress. However, considering the characteristics of the event camera itself, the designed neural network should consider both running time and reconstruction accuracy.

## 3. E2VIDX method

This section outlines the specific implementation process of E2VIDX. To feed a stream of events into a neural network, we need to encode the data stream. The encoded tensors are, then, fed into E2VIDX, a convolutional neural recurrent network for training. To efficiently fit the model with the training data set, a convenient and efficient loss function is also designed.

### 3.1. Event encoding

The event camera output is in the form of event streams, as shown in Equation 1.


(1) $$\begin{array}{l} e_{i}\left(p,t\right)=\sigma_{i}^{p}c\delta \left(t-t_{i}^{p}\right),i\in 1,2,3\ldots \end{array}$$

Here, we denote σ∈{−1, 1} as polarity, *p* = (*x, y*) as event coordinates, *c* as the contrast threshold that triggers an event, and δ as the Dirac delta function. To enable the convolutional recurrent neural network to process the event stream, it is essential to encode the event stream into a fixed-size spatiotemporal tensor. The event stream is partitioned into groups based on their timestamp order, with each group containing *N* events, denoted as ε_*k*_ = {*e*_*i*_}, *i*∈[0, *N*−1]. This encoding transforms the event stream into a spatiotemporal stereo tensor, which serves as the input. For each event group denoted as ε_*k*_, we quantize the time interval as $\Delta T=t_{N-1}^{k}-t_{0}^{k}$ and distribute it across *B* time channels. Within each event *e*_*i*_, its polarity is associated with the same spatial location and its two closest time channels in the group *E*_*k*_, as shown in Equation 2.


(2) $$\begin{array}{l} \text{E}\left(x_{l},y_{m},t_{n}\right)=\underset{x_{i}=x_{l}y_{i}=y_{m}}{\sum }p_{i}\max\left(0,1-|t_{n}-t_{i}^{*}|\right) \end{array}$$

where $t_{i}^{*}≜\frac{B-1}{\Delta T}\left(t_{i}-t_{0}\right)$ is the benchmark time after standardization. Like other methods Wang et al. (2019), Rebecq et al. (2019a,b), Scheerlinck et al. (2020), Cadena et al. (2021), we also set *B* as 5 for our experiment.

### 3.2. Network design

**Figure 2:**
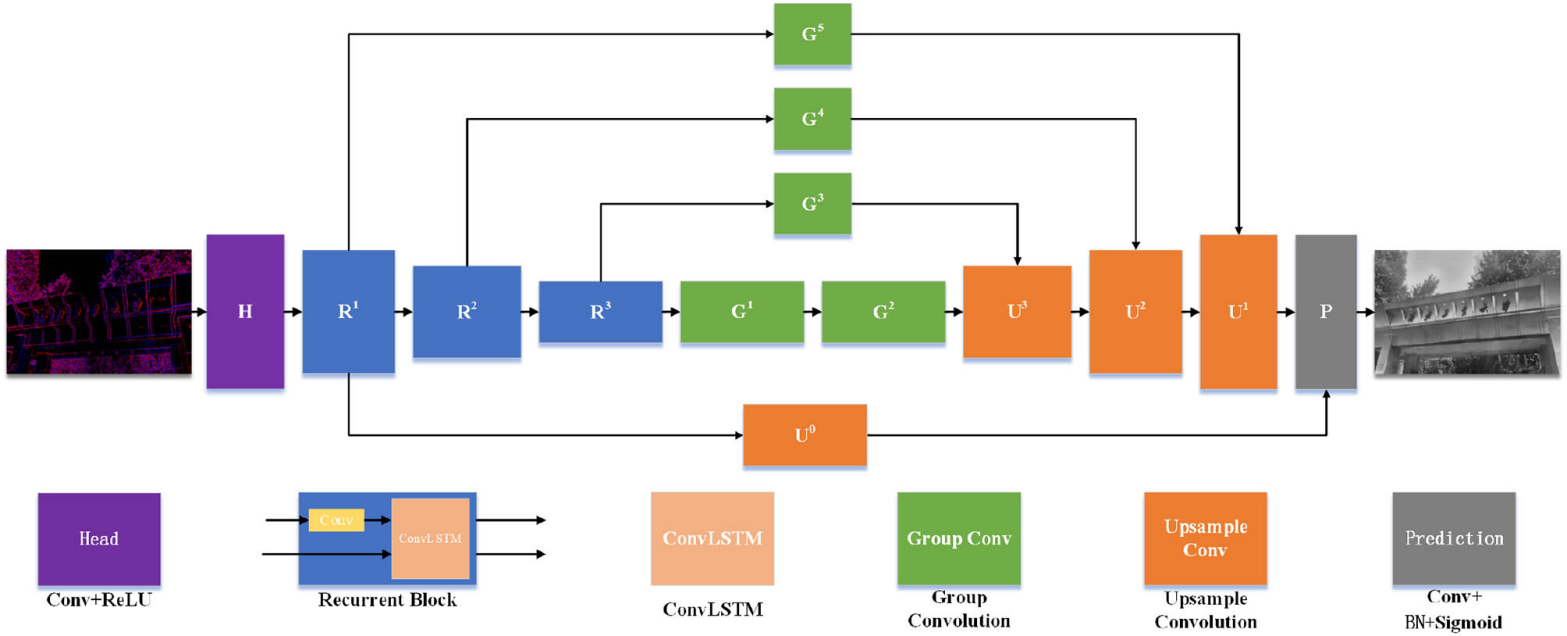
The network structure of E2VIDX. The U-Net network structure is used, which is divided into the downsampling part and upsampling part, and new feature fusion is added at the same time. In the downsampling part, ConvLSTM is used to fuse the previous reconstruction state information, and subpixel convolution is used to avoid checkerboard artifacts in the upsampling part.

The overall structure of E2VIDX is similar to U-net, which is divided into the head, body, and prediction layers, as shown in Figure 2. The body layer comprises the downsampling part and the upsampling part. Unlike E2VID, we add group convolution branch to downsampling layer which helps in feature fusion during upsampling. The original ResBlock is replaced by group convolution, and by observing the output of each layer in training, part of the input of the actual output layer is modified for better low-level and high-level feature fusion. Meanwhile, learnable sub-pixel convolution is used in the upsampling part.

#### 3.2.1. Head

After event encoding, the neural network gets fixed-size tensors with five channels as input. The primary purpose of the head layer is to expand the number of channels to facilitate subsequent feature extraction. The kernel size used in this layer is 3.

#### 3.2.2. Body

**Figure 3:**
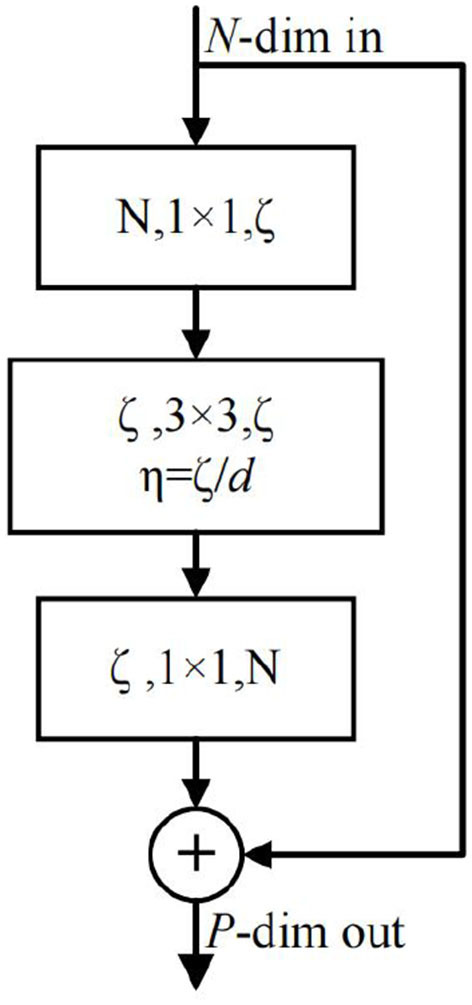
Schematic diagram of group convolution. Compared with the original ResBlock, we group the input channels and perform operations on each group before the confluence.

The body part is the central part of the whole network, which completes the feature extraction and fusion. The downsampling part consists of three recurrent convolution modules with ConvLSTM (Shi et al., 2015). Each convolutional block consists of CBR (Conv+BatchNorm+ReLU) and ConvLSTM modules. The purpose of using ConvLSTM is to preserve the previous state information, which is used to update the current state in combination with the current input. Therefore, the convolutional block operation feeds the input into the CBR and then updates the output as a partial input to the ConvLSTM. The size of the convolution kernel in each convolution block is 5, the stride and padding are 2, and the number of output channels is twice of the input. Therefore, the width and height of the tensor are halved, and the number of channels is doubled for each convolution block. We also feed the output of each convolutional block into a branch, each of which is made up of group convolutions (Xie et al., 2017). We use group convolution instead of the original ResBlock because not only they can effectively reduce the number of parameters but also can speed up the training. After the bottom layer sampling, two layers of group convolutions are connected, aiming to extract the most abstract features fully. The group convolution we employ is shown in Figure 3. The parameters involved are the input dimension *N*, the depth of the channel *d* in each group, the group number η, the total number of group convolution channels ζ, and the number of output channels *P*. In this study, our relationship between these parameters is: *N* = 2ζ = 8η, ζ = *dη*.

**Figure 4:**
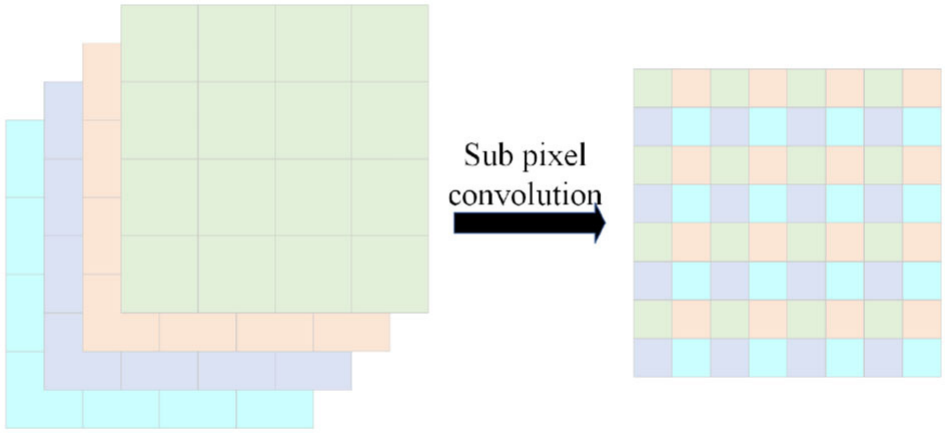
Schematic diagram of subpixel convolution. The expansion is realized by arranging the identical coordinate position tensors on the channel.

The next step is followed by three upsampling layers, where the input of each upsampling layer is the output of the corresponding downsampling layer processed by the group convolution branch and the output of the previous upsampling layer. Traditional upsampling is achieved by unlearnable methods such as linear interpolation; however, we use subpixel convolution Shi et al. (2016) to replace the original interpolation. The schematic of the sub-pixel convolution is shown in Figure 4. We use sub-pixel convolution for upsampling on each layer because it can effectively decrease the number of arguments (channel count will become $\frac{1}{r^{2}}$, where *r* is the upsampling factor). Additionally, the parameters of the sub-pixel convolution are learnable; its weight changes during training can effectively eliminate checkerboard artifacts (Shi et al., 2016).

#### 3.2.3. Prediction

**Figure 5:**
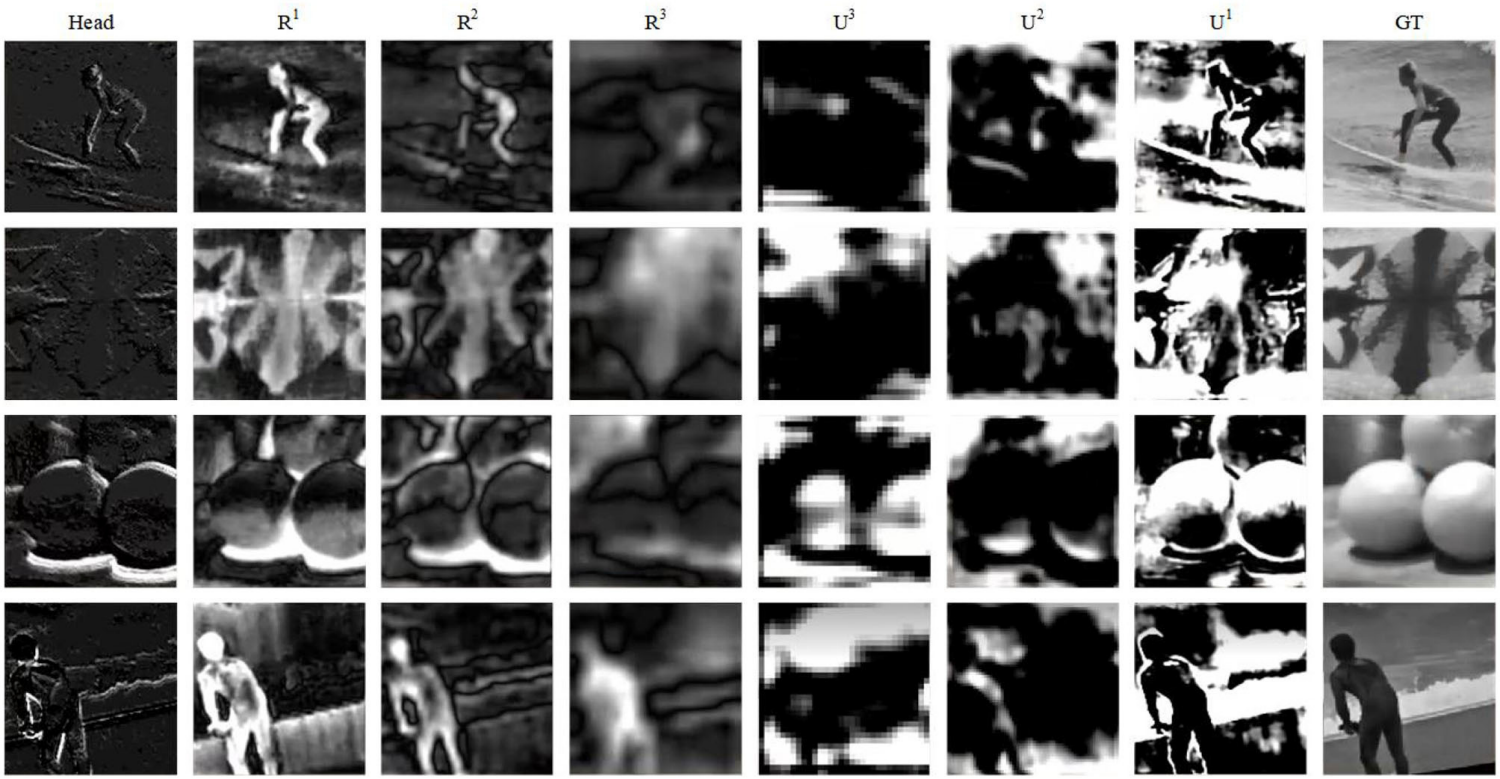
Visualization of each layer of the network.

At the end, the network is the prediction layer, which for each pixel, predicts a value between 0 and 1. The input to the prediction layer is the sum of the upsampled output of *R*^1^ and *U*^1^. The expected inputs to the prediction layer are deep features and shallow features with good quality. Figure 5 shows the visual output of each layer in the network. The head layer's output is sparse, meaning the shallow features are insufficient, so we consider *R*^1^ as representative. *U*^1^ is the output after upsampling iteration, which has higher level feature properties and is used as a deep feature representative. After getting the input of the prediction layer, it is convolved with a convolution kernel of size 1 × 1, then fed into the BN layer. Finally, the output is obtained by the Sigmoid activation function.

### 3.3. Loss fuction

To obtain a reconstructed image with rich feature information, the loss function consists of two parts. The first part LPIPS (Zhang et al., 2018) is used to measure the high-level features of the image. The second part SSIM (Wang et al., 2004) is to calculate the low-level features. SSIM measures the similarity between two images, mainly judged by focusing on the similarity of edges and textures. Its calculation formula is as follows:


(3) $$\begin{array}{l} \mathrm{SSIM}\left(X_{1},X_{2}\right)=L\left(X_{1},X_{2}\right)\times C\left(X_{1},X_{2}\right)\times S\left(X_{1},X_{2}\right) \end{array}$$

where *X*_1_ and *X*_2_ represent two images, *L* represents brightness similarity, *C* represents contrast similarity, and *S* represents structure score. *L*, *C*, and *S* are, respectively, calculated as follows:


(4) $$\begin{array}{l} \begin{array}{cc} L\left(X_{1},X_{2}\right) & =\frac{2u_{X_{1}}u_{X_{2}}+C_{1}}{u_{X_{1}}^{2}+u_{X_{2}}^{2}+C_{1}}\\ C\left(X_{1},X_{2}\right) & =\frac{2\sigma_{X_{1}}\sigma_{X_{2}}+C_{2}}{\sigma_{X_{1}}^{2}+\sigma_{X_{2}}^{2}+C_{2}}\\ S\left(X_{1},X_{2}\right) & =\frac{\sigma_{X_{1}X_{2}}+C_{3}}{\sigma_{X_{1}}\sigma_{X_{2}}+C_{3}} \end{array} \end{array}$$

In the above, *u*_*X*_1__ and *u*_*X*_2__ represent the mean of images *X*_1_ and *X*_2_, σ_*X*_1__ and σ_*X*_2__ represent the standard deviation, and σ_*X*_1_*X*_2__ represents the covariance, respectively. *C*_1_, *C*_2_, and *C*_3_ are constants used to avoid division by 0. Specifically, *C*_1_ = 0.01, *C*_2_ = 0.03, and *C*_3_ = 0.015.

To increase the similarity of the two images, it is also necessary to make their error in high-level feature expression as small as possible; here, LPIPS is used to achieve that goal. LPIPS uses a VGG19 (Simonyan and Zisserman, 2014) network trained in the MS-COCO dataset to let two images pass through the network and calculate the difference between the output value of each layer of the network.


(5) $$d\left(X_{1},X_{2}\right)=\sum_{l}\frac{1}{H_{l}W_{l}}\sum_{h,w}{\left\|w_{l}⊙\left({\hat{Y}}_{1hw}^{l}-{\hat{Y}}_{2hw}^{l}\right)\right\|}_{2}^{2}$$

where *d* is the mean difference between *X*_1_ and *X*_2_. Feature pairs are extracted from the *l* layer and unit normalized in the channel dimension. *w*_*l*_ is the scaling factor, ⊙ stands for the inner product, and Ŷ is the output of the corresponding layers. The final loss function is as follows:


(6) $$\begin{array}{l} L=\mathrm{SSIM}\left(X_{1},X_{2}\right)+d\left(X_{1},X_{2}\right) \end{array}$$

### 3.4. Training

Since the ground truth is not easy to obtain when the actual event camera is used to make the dataset, all the datasets used by the mainstream methods (Rebecq et al., 2019a,b; Scheerlinck et al., 2020; Cadena et al., 2021) are generated in the simulator. For fair evaluation, this study also uses the same dataset. Based on the MS-COCO dataset, the ECOCO dataset (Lin et al., 2014) is used. The event simulator ESIM (Rebecq et al., 2018) is used to map and generate the corresponding event stream and regular image. The image size used in the simulator is 240 × 180 pixels. The simulator was used to generate 1,000 sequences, each event lasting for 2 s, 950 sequences were randomly selected as the training set, and the rest were used as the test set. For all event streams, normal distribution random noise with a mean of 0.18 and a standard deviation of 0.03 are added. The purpose of this is to mimic the noise of the actual camera itself and avoid over-fitting during training, which leads to poor reconstruction results in natural conditions.

During training, the data were randomly flipped [-20°, 20°], randomly flipped horizontally, and cropped to 128 × 128 size to increase the dataset. Our experiments are conducted on the Ubuntu 18.04 LTS operating system using CUDA 11.0, Python 3.8, and PyTorch 1.3.0. The hardware setup included NVIDIA GTX 1080 (8GB), 64GB of RAMs, and an Intel i7-12700 CPU. The epoch is 200, the batch size is 4, ADAM (Kingma and Ba, 2014) optimizer is used, the maximum learning rate is 5 × 10^−4^, and warm up learning strategy is adopted.

## 4. Experiment and analysis

In this section, we qualitatively evaluate E2VIDX against current mainstream methods and then apply it in practice.

### 4.1. Reconstructed image evaluation

To measure the accuracy of each method, we use the same dataset as the previous study (dynamic_6dof, boxes_6dof, poster_6dof, office_zigzag, slider_depth, and calibration). The dataset was taken indoors under six scenarios. It contains variable speed-free motion with six degrees of freedom and linear motion with only one degree of freedom. The camera model used in the dataset is DAVIS240C, which can output event streams and frame images of 240 × 180 size. Each reconstructed image is matched with the frame image with the closest timestamp. MSE, SSIM, and LPIPS of the two images were calculated as evaluation metrics. The qualitative indicators in each dataset are shown in Table 1. We use sub-pixel convolution and group convolution, which means a boost on the low-level features of the image. Therefore, the obtained reconstructed image has better performance in SSIM and MSE. SPADE-E2VID adds weight to the LPIPS term in the loss function, so it performs best on LPIPS. In addition to that on LPIPS, our method performs better than both E2VID and FireNet.

**Table 1:** The evaluation index scores of the reconstruction results.

**Datasets**	↑**SSIM**				↓**LPIPS**				↓**MSE**			
	**E2VID**	**FireNet**	**SPADE-E2VID**	**E2VIDX Ours**	**E2VID**	**FireNet**	**SPADE-E2VID**	**E2VIDX Ours**	**E2VID**	**FireNet**	**SPADE-E2VID**	**E2VIDX Ours**
dynamic_6dof	0.3841	0.3737	0.3742	**0.4256**	0.3621	0.3348	**0.3208**	0.3472	0.1560	0.1457	0.1073	**0.0759**
boxes_6dof	0.5693	0.5143	0.5537	**0.5700**	0.3111	0.3429	**0.2921**	0.3023	**0.0414**	0.0546	0.0446	0.0426
poster_6dof	**0.5616**	0.5420	0.5537	0.5567	0.2916	**0.2860**	0.2877	0.3074	0.0638	**0.0487**	0.0565	0.0624
office_zigzag	0.4474	0.4261	0.4479	**0.4635**	0.3208	0.3393	**0.3031**	0.3209	0.0739	0.0813	0.0560	**0.0515**
slider_depth	0.2821	0.2683	0.2672	**0.3006**	0.4095	0.4097	0.4077	**0.3679**	0.1035	0.0824	0.0803	**0.0696**
calibration	0.3795	0.3613	0.3813	**0.3889**	0.3544	0.3138	**0.2598**	0.2987	0.0698	0.0617	**0.0543**	0.0567
**Mean**	0.4373	0.4143	0.4297	**0.4504**	0.3416	0.3378	**0.3118**	0.3241	0.0847	0.0791	0.0665	**0.0598**

The higher the SSIM score, the better and the lower the LPIPS and MSE scores, the better. The bold values show that the score is the best compared with other methods.

**Figure 6:**
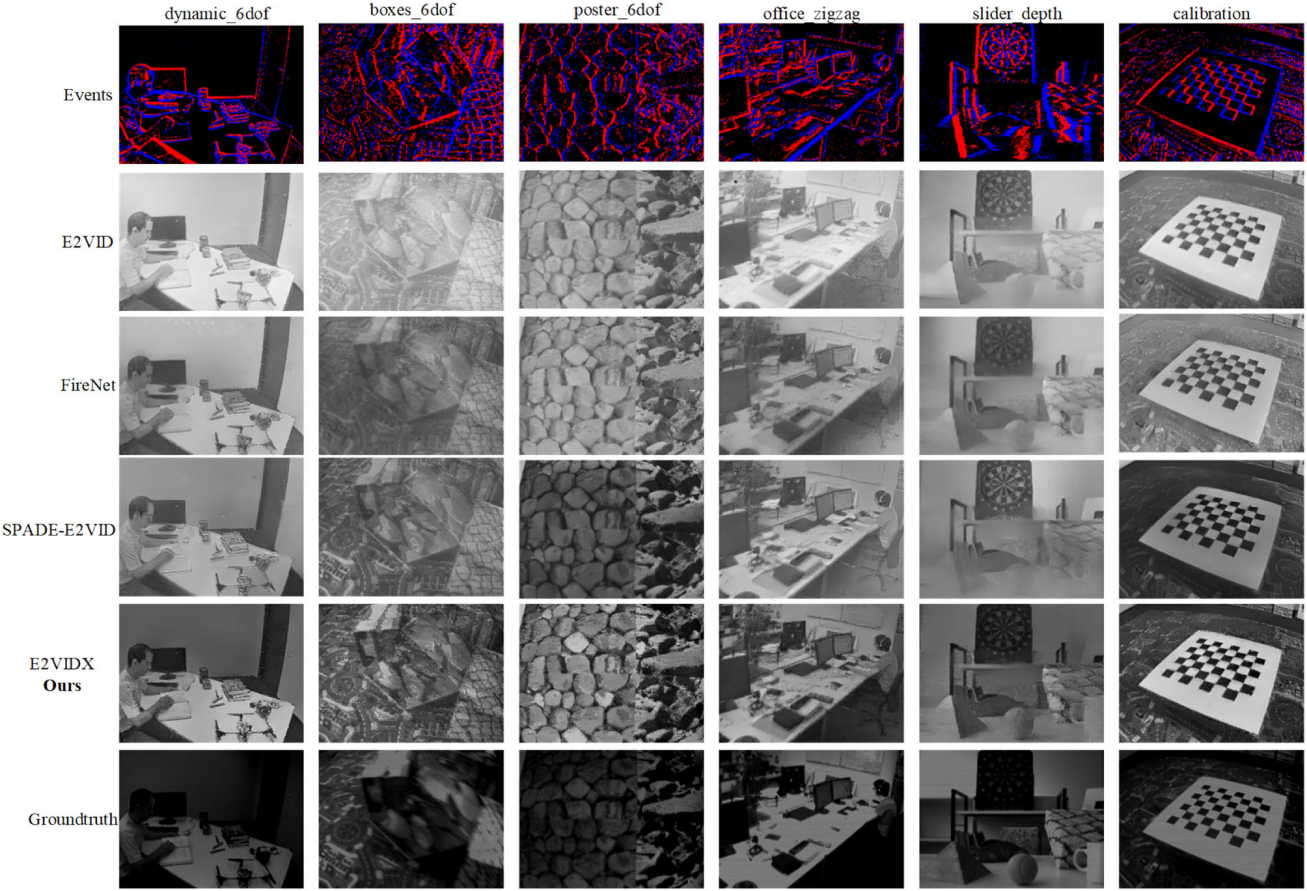
Comparison of reconstruction results.

Figure 6 shows the reconstruction results of various methods. The reconstructed images of E2VID and FireNet have a white foreground, causing the deviation of color saturation. SPADE-E2VID has a good performance in reconstruction images, but it needs the previous reconstruction image as input; the accumulated error often cannot be eliminated. Our method performs better in terms of color saturation and contrast and achieves the best performance in terms of SSIM and MSE.

In addition, we calculate the time required for various methods. We made a dataset at each of the four resolutions and averaged three tests of each method using GPU and CPU. The results are presented in Table 2. FireNet has the lowest time required due to its lightweight network. However, its reconstruction accuracy is not high. Compared with E2VID and SPADE-E2VID, our method is approximately 10% and 60% faster, respectively, and has the best accuracy. Therefore, FireNet is only necessary when computing power is very limited. Our proposed method can improve the reconstruction accuracy while ensuring as delay as possible.

**Table 2:** Timing Performance (ms).

**Methods**	**Resolution**	**E2VID**	**FireNet**	**SPADE-E2VID**	**E2VIDX Ours**
**GPU**	240 × 180	8.02	**2.81**	22.02	8.19
	480 × 320	22.28	**9.46**	70.48	20.65
	640 × 480	42.70	**16.86**	138.44	38.52
	1280 × 720	123.42	**51.15**	375.42	108.72
**CPU**	240 × 180	86.62	**13.98**	294.04	63.18
	480 × 320	296.53	**65.28**	1042.35	242.59
	640 × 480	588.39	**150.28**	2210.71	496.44
	1280 × 720	1870.22	**581.61**	6672.57	1596.67

The GPU is NVIDIA GTX 1080 (8GB) and the CPU is Intel i7-12700. The bold values show that the score is the best compared with other methods.

### 4.2. Ablation study

**Figure 7:**
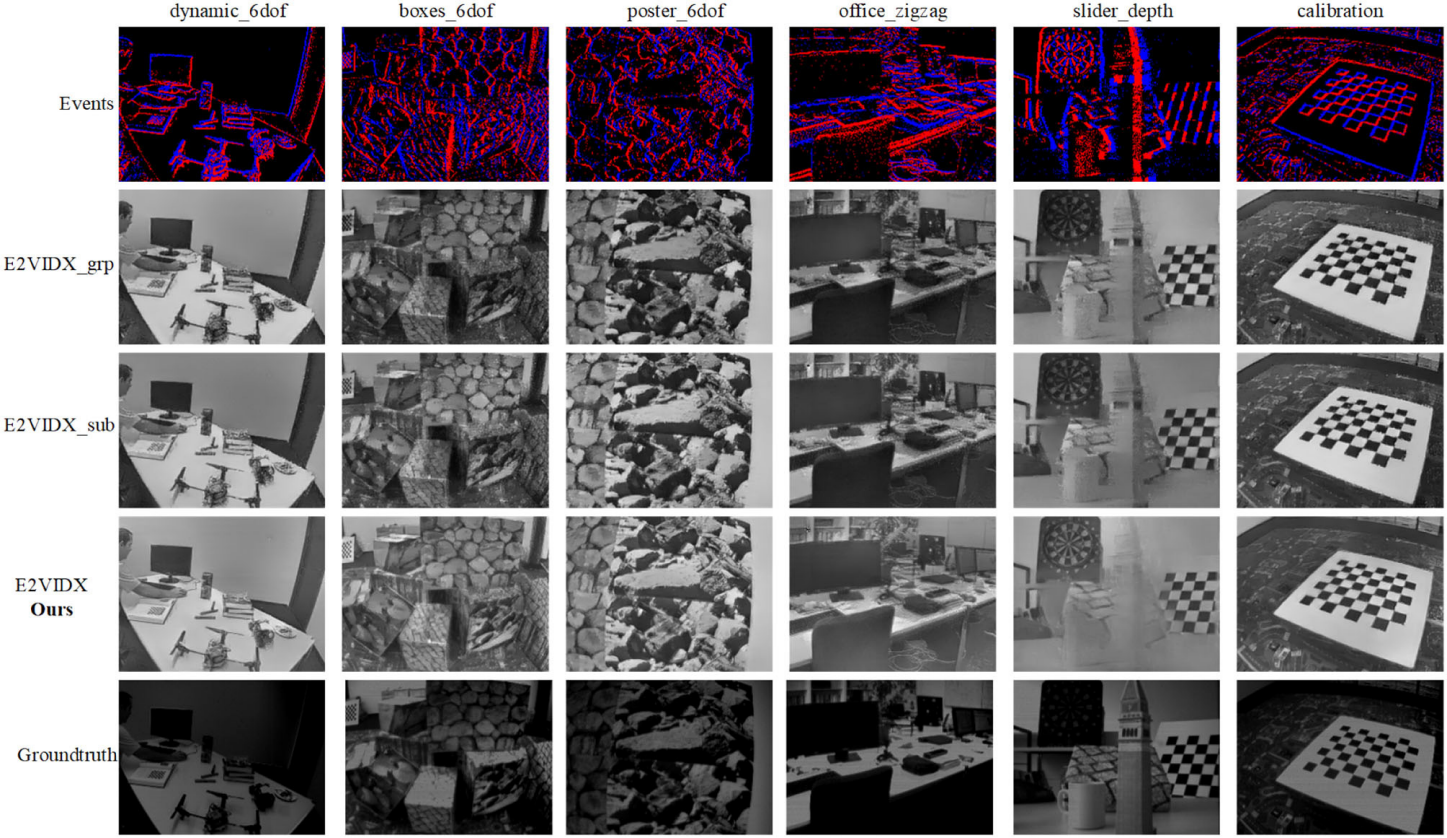
Reconstruction results of ablation study. E2VIDX_grp represents the use of group convolution only, and E2VIDX_sub represents the use of subpixel convolution only.

To demonstrate the effectiveness of the network design, we designed an ablation study. Experiments are conducted to test the used group convolution and subpixel convolution. For the same hardware environment, keeping other network parameters same, the network is trained for the same epoch. The test results are shown in Table 3, and the representative reconnection results are shown in Figure 7.

**Table 3:** Score of ablation study evaluation index.

**Datasets**	↑**SSIM**		↓**LPIPS**		↓**MSE**	
	**E2VIDX_grp**	**E2VIDX_sub**	**E2VIDX_grp**	**E2VIDX_sub**	**E2VIDX_grp**	**E2VIDX_sub**
dynamic_6dof	0.3919	0.4015	0.3683	0.3301	0.1376	0.1069
boxes_6dof	0.5595	0.5711	0.3140	0.3142	0.0450	0.0411
poster_6dof	0.5630	0.5603	0.3072	0.3184	0.0642	0.0632
office_zigzag	0.4519	0.4639	0.3349	0.3242	0.0676	0.0547
slider_depth	0.2880	0.3023	0.3896	0.3762	0.0817	0.0739
calibration	0.3691	0.3978	0.3142	0.3002	0.0645	0.0557
**Mean**	0.4372	0.4495	0.3380	0.3272	0.0768	0.0659

E2VIDX_grp represents the use of group convolution only, and E2VIDX_sub represents the use of subpixel convolution only.

It can be observed from the table that the two groups of ablation study have a certain degree of decline in the three indices compared with E2VIDX. Among them, the group of experiments without group convolution score better in the evaluation indices, indicating that subpixel convolution has a significant influence on our model. It is also noted that even the ablation studies perform better than E2VID, indicating that we have appropriately chosen our network design, loss function, and data processing. From the perspective of images, the images reconstructed by the ablation study are close to E2VIDX in terms of color and contrast, which can recover the results of perceptual solid perception. However, the images of E2VIDX_grp are missing in detail (burrs appear on the edges of the objects).

### 4.3. Applications

In this section, the reconstructed images are mainly used for various computer vision applications, and three popular visual application experiments are mainly carried out for task difficulty: image classification, object detection, and instance segmentation. The hardware and software platforms used in this section are the same as those mentioned in Section 3.4.

#### 4.3.1. Image classfication

**Figure 8:**
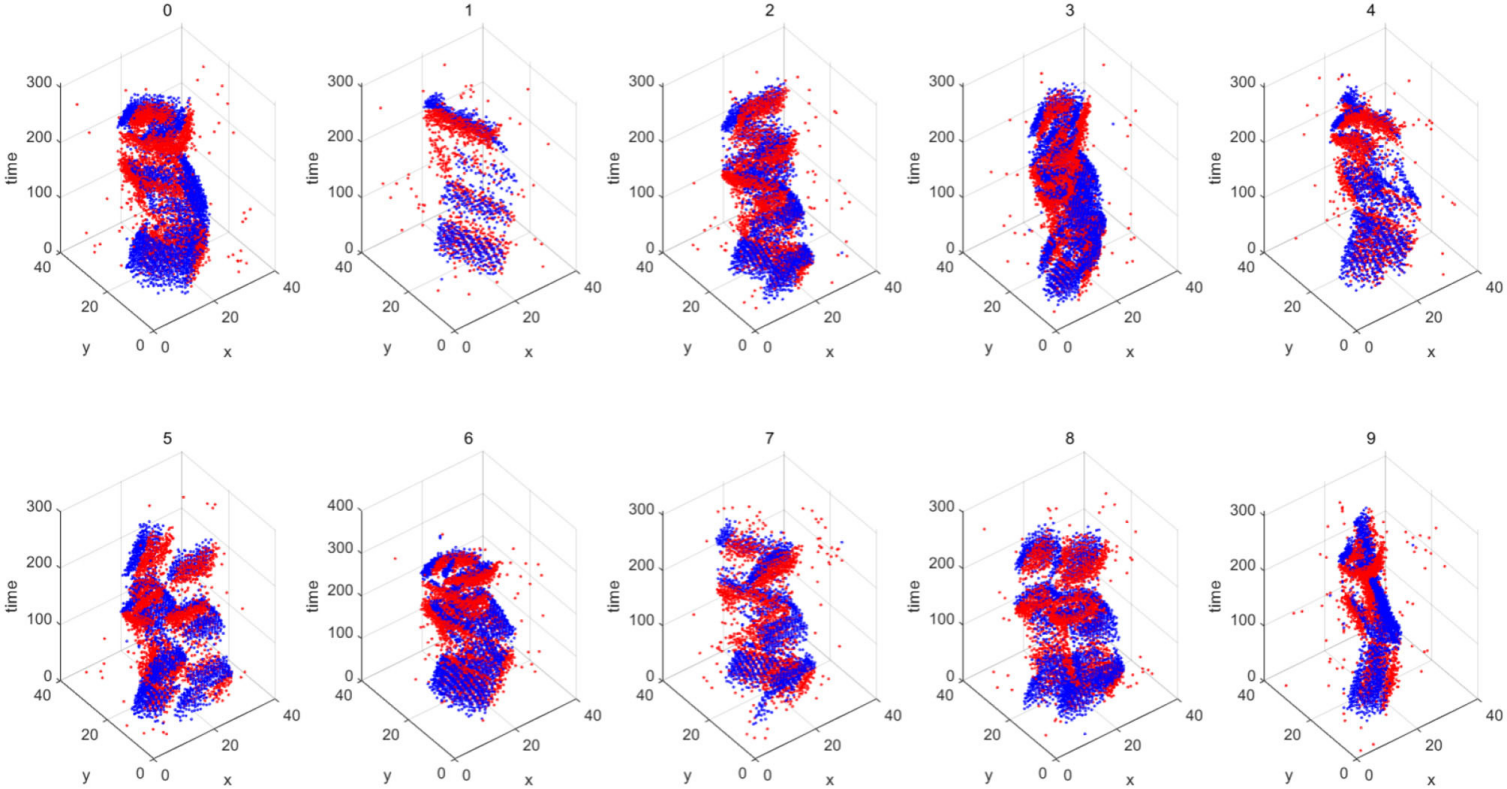
Overview of the N-MNIST dataset. The blue point clouds represent negative polarity and the red point clouds represent positive polarity. x and y are two-dimensional representations of the space.

**Figure 9:**
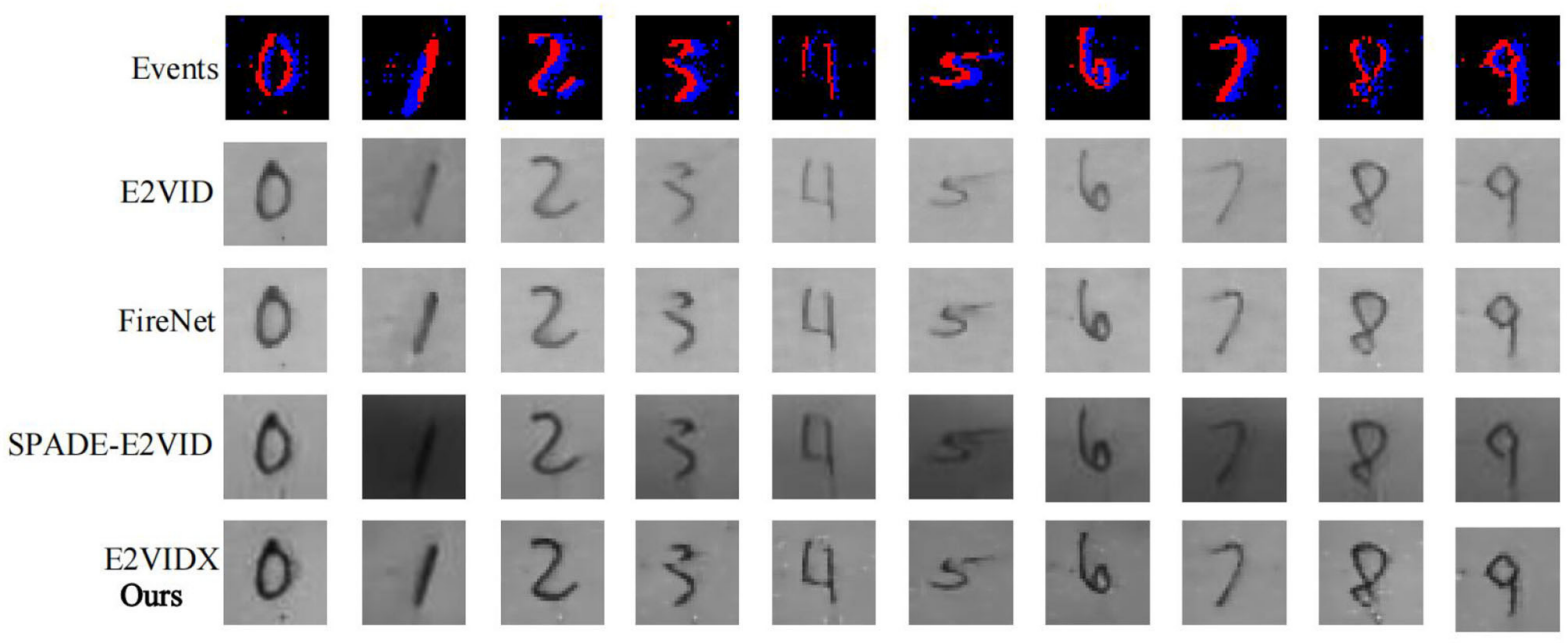
N-MNIST dataset reconstruction results.

Image classification is one of the basic tasks in computer vision, which aims to identify the objects in the image. With the recent advancements in neural networks, this task has been well solved (the accuracy can even exceed the human eye Russakovsky et al., 2015). The datasets in this domain include MNIST (LeCun et al., 1998) and CIFAR-10 (Krizhevsky and Hinton, 2009), which contain regular images and labels. Compared with the previous image classification, the image classification task in this section is carried out under the dataset captured by the event camera. The Neuromorphic-MNIST (N-MNIST) dataset (Orchard et al., 2015) is a “Neuromorphic” version of the MNIST dataset. It is captured by mounting an Asynchronous Time-based Image Sensor (ATIS) (Posch et al., 2010) on the motorized head unit and allowing the sensor to move while viewing the MNIST dataset on the LCD (Figure 8). To fully demonstrate the reliability of image reconstruction, we use LeNet5 (LeCun et al., 1998) to train on the MNIST dataset to obtain the corresponding weight file and then directly use this file to classify and recognize the image reconstructed by the image reconstruction algorithm on N-MNIST. The corresponding reconstruction results are shown in Figure 9, and the classification accuracy is shown in Table 4.

**Table 4:** Classification accuracy of N-MNIST dataset.

	**E2VID**	**FireNet**	**SPADE-E2VID**	**E2VIDX Ours**
Mean accuracy	85.78%	85.92%	84.03%	**86.71**%

The bold values show that the score is the best compared with other methods.

From Figure 9, it can be observed that the reconstruction results of these four methods can accurately recover the handwritten numbers, among which the images of E2VID and FireNet are still slightly white, resulting in insufficient color. SPADE-E2VID needs more time to initialize at the beginning of the reconstruction result because the input needs the output from the previous step. The proposed method (E2VIDX) can provide high-quality reconstructed images. It is worth mentioning that although our LeNet5 is trained on the MNIST dataset, the classification accuracy of N-MNIST dataset is more than 84% (the accuracy of our proposed E2VIDX is the highest 86.71%). This shows that the reconstruction method is reliable and can recover the corresponding feature information.

#### 4.3.2. Object detection

Object detection technology has always been one of the challenging fields in computer vision. The object detection task is to automatically identify the object contained in the input image and return its target pixel coordinates and target category. Object detection technology based on deep learning has been extensively researched. Up to now, there have been many excellent object detection algorithms, such as R-CNN series (Girshick, 2015; Ren et al., 2015), YOLO series (Redmon et al., 2016; Redmon and Farhadi, 2017, 2018), and SSD series (Liu et al., 2016; Li and Zhou, 2017; Yi et al., 2019). This section aims to prove the reliability of each reconstruction algorithm. The popular YOLOv5 (Zhang et al., 2022) object detection algorithm is adopted to detect the reconstructed image. The task in this section is still using transfer learning as mentioned in the previous section. The model trained on the conventional image is directly used to detect and reconstruct the image. Specifically, YOLOv5s that has been trained on the COCO dataset is used for detection.

**Figure 10:**
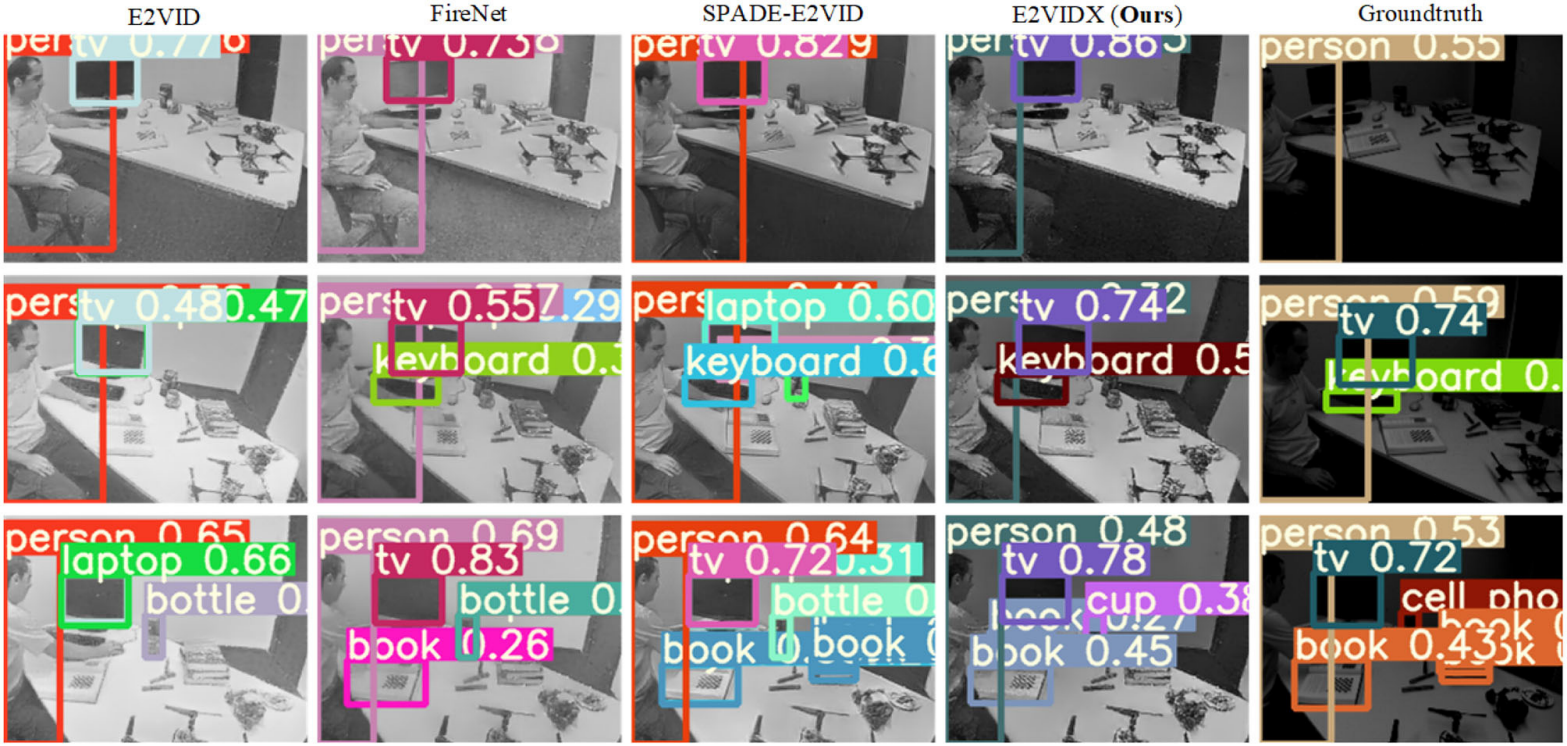
YOLOv5 for reconstructed image detection.

Since there are no corresponding labels in the ECOCO dataset, we can only present qualitative experimental results, as shown in Figure 10. It can be observed from the figure that all reconstruction methods can directly identify the main object, but there are differences in the specific class and confidence. E2VIDX's reconstructed image detection results are improved in confidence compared with the frame images, which indicates that our recovered images have strong interpretability. The detection results of E2VIDX and SPADE-E2VID are better than E2VID and FireNet in object recognition and confidence, especially in the recognition of small objects, such as books.

#### 4.3.3. Instance segmentation

**Figure 11:**
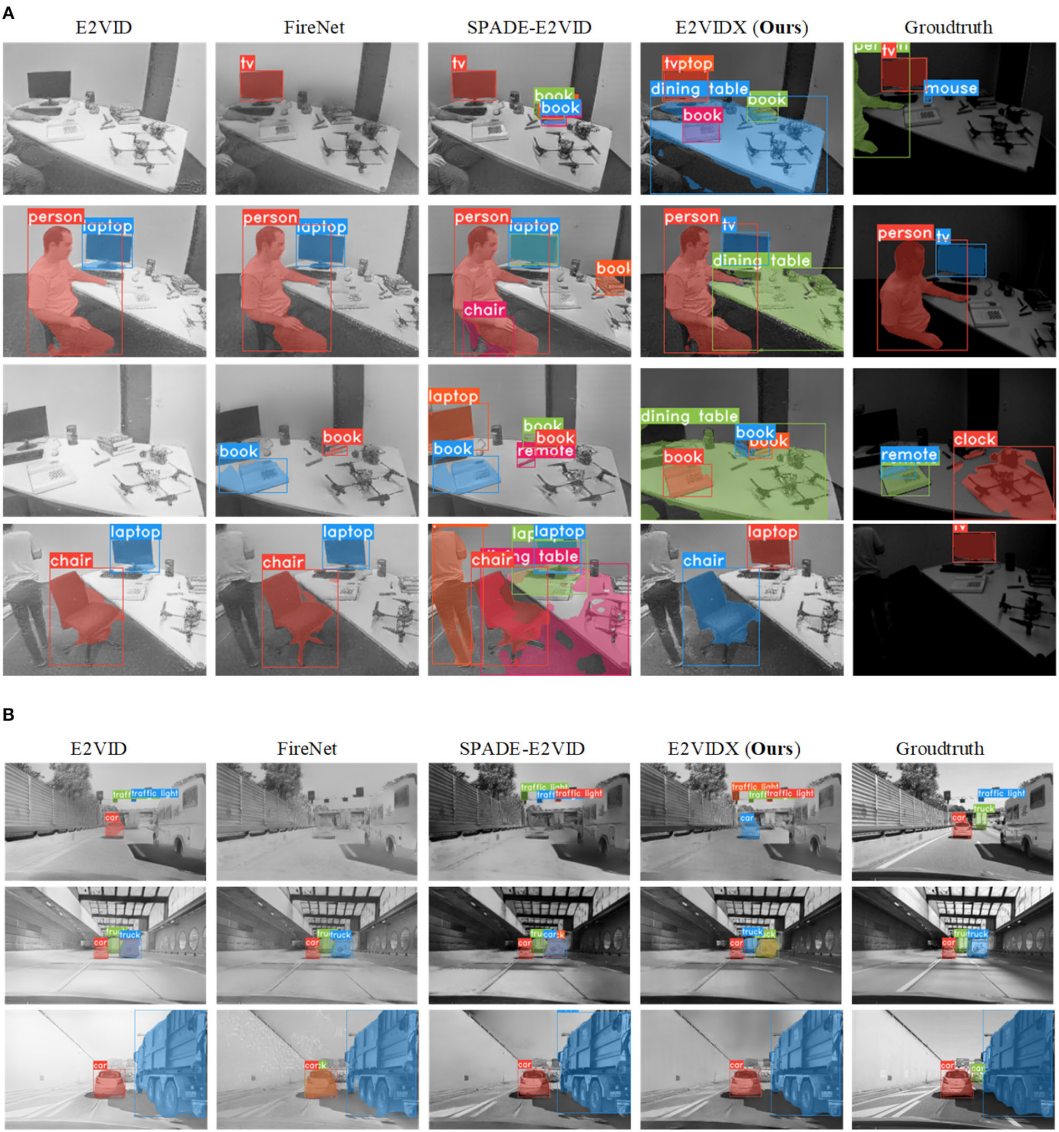
YOLACT for reconstructed image instance segmentation. **(A)** Instance segmentation of reconstructed images of indoor scenes. **(B)** Instance segmentation of reconstructed images of outdoor scenes.

As one of the difficult visual tasks, instance segmentation, is also the focus of current research. Instance segmentation classifies the image pixel-by-pixel, so it requires high quality of the image itself. In this section, we use the YOLACT (You Only Look At Coefficients) (Bolya et al., 2019) instance segmentation model to conduct experiments and also use the weight files trained under the regular camera dataset to directly segment our reconstructed image. The previously used datasets were all taken indoors, so the reconstruction of outdoor scenes is added in this section. The specific scene is a motor vehicle on the highway. After taking frame images with Huawei P20 Pro, VID2E (Hu et al., 2021) is used to transform them into event streams, and then, reconstruction is performed. The segmentation results of our reconstruction results are shown in Figure 11.

For indoor scenes, it can be observed that the segmentation effect of E2VIDX is more continuous and accurate compared with other methods. Our method can outline most objects by pixels. In comparison, other methods do not achieve the same performance because the reconstruction results are not ideal and thus can lead to missed detection or false detection. Due to the insufficient illumination conditions, the false detection rate for instance segmentation in frame images is high.

For outdoor scenes, E2VIDX performs image reconstruction equally well, and the reconstructed images are highly consistent with the high-quality original images. The segmentation of the two images (original and reconstructed) is almost the same, indicating that the recovered image has similar characteristics with the high-quality frame image. The outdoor segmentation results of other methods generally perform well but occasionally have misdetection.

## 5. Conclusion

In this study, we propose a novel approach named E2VIDX for the field of event camera-based image reconstruction. Specifically, our study proposes: (1) the optimization of the original network structure to strengthen the feature fusion of deep and shallow layers; (2) use of group convolution and sub-pixel convolution to further strengthen the model and the related ablation study to verify its effectiveness. (3) A simple loss function, which is optimized from the semantic and low-level features of the image. Furthermore, we evaluate the reconstructed results in practical vision applications, including image classification, object detection, and instance segmentation. We conduct comprehensive quantitative and qualitative experiments to assess the performance of our approach. Through rigorous experimentation, E2VIDX surpasses the current state-of-the-art methods. When compared with E2VID, our approach exhibits notable improvements, including a 1.3% increase in SSIM, a reduction of 1.7% in LPIPS, a 2.5% decrease in MSE, and a 10% reduction in inference time. We also optimize the model size, reducing it from 32.1MB to 42.9MB. After conducting a series of comparative experiments, we demonstrate that E2VIDX boasts enhanced robustness, enabling direct application of the reconstructed image data. This effectively narrows the gap between conventional computer vision and biomimetic vision. In future, our research will primarily concentrate on the development of a lightweight network structure. We aim to enhance the efficiency of feature extraction by integrating advanced attention mechanisms into our model.

## Data availability statement

The original contributions presented in the study are included in the article/supplementary material, further inquiries can be directed to the corresponding author.

## Author contributions

XH: Methodology, Writing—original draft. FZ: Methodology, Writing—review & editing. DG: Writing—review & editing. TT: Software, Visualization, Writing—review & editing. WZ: Software, Visualization, Writing—review & editing.
